# Impact of parental psychological intervention on long-term outcomes following bariatric surgery: a retrospective cohort study

**DOI:** 10.3389/fsurg.2026.1909015

**Published:** 2026-07-28

**Authors:** ChenXing Cui, Kai Ma

**Affiliations:** Department of General Surgery, Shanxi Bethune Hospital, Taiyuan, Shanxi, China

**Keywords:** bariatric surgery, family dynamics, obesity, parental psychological intervention, retrospective study

## Abstract

**Background:**

The efficacy of bariatric surgery is often compromised by psychosocial factors within the family environment. Clinical observations suggest that parents of patients with obesity frequently exhibit maladaptive psychological traits, such as anxiety and excessive control, which may hinder long-term postoperative weight loss.

**Methods:**

We conducted a retrospective cohort study involving 200 patients who underwent bariatric surgery (Laparoscopic Sleeve Gastrectomy or Roux-en-Y Gastric Bypass) at our institution between January 2018 and January 2024. Patients treated from 2018 to early 2021 (*n* = 100) comprised the Control Group (standard surgical care). Patients treated from 2021 to 2024 (*n* = 100) comprised the Observation Group, who received standard surgical care plus a structured, multi-session psychological intervention for their parents, based on Cognitive Behavioral Therapy (CBT) and Family Systems Therapy. The primary outcomes were the percentage of excess weight loss (%EWL) and weight regain rate at 24 months postoperatively.

**Results:**

Baseline demographics and preoperative BMI were comparable between groups. At the 24-month follow-up, the Observation Group demonstrated significantly superior outcomes. The mean %EWL was 78.2% ± 6.1% in the Observation Group vs. 55.1% ± 7.5% in the Control Group (*P* < 0.001). The weight regain rate was significantly lower in the Observation Group (4.2% vs. 18.5%, *P* < 0.01). Pearson correlation analysis revealed a significant positive correlation between the reduction in parental control scores and patient dietary adherence scores at 24 months (r = 0.86, *P* < 0.001) within the Observation Group.

**Conclusion:**

Integrating targeted psychological intervention for parents into bariatric surgery protocols is associated with significantly improved long-term weight loss and reduced weight regain. Addressing maladaptive family dynamics, specifically parental anxiety and controlling behaviors, appears to be a crucial component for the durable success of metabolic surgery. Due to the quasi-experimental design, these findings warrant confirmation in prospective, randomized controlled trials.

## 1 Introduction

The global prevalence of obesity has reached pandemic proportions, necessitating effective therapeutic strategies. Bariatric surgery is currently the most effective treatment for severe obesity and its metabolic comorbidities (1, 2). However, long-term success is not guaranteed; a significant subset of patients experience weight regain or suboptimal weight loss years post-intervention (3, 4). Emerging evidence suggests the etiology of obesity extends beyond physiological dysregulation to include complex psychosocial and environmental factors, with the “family system” playing a pivotal role (5).

In our clinical practice, we observed a distinct pattern among many patients: their parents often exhibited pronounced psychopathological traits, including anxiety (manifested as persistent fear of the patient undereating) and excessive behavioral control. This phenomenon can be conceptualized as “enmeshment” or a “maladaptive symbiotic relationship,” where parents unconsciously project anxieties onto the child, using food for emotional regulation or control (6). Such dynamics undermine patient autonomy and self-efficacy, critical for adhering to strict post-surgical dietary and lifestyle modifications. Despite the importance of psychosocial support, current guidelines rarely address the mental health of the parents or caregivers (7).

To address this gap, we introduced a multidisciplinary intervention providing psychological therapy to parents of patients undergoing bariatric surgery starting in 2021. This retrospective study evaluates the association between this intervention and patient postoperative outcomes.

## 2 Materials and methods

### 2.1 Study design and participants

We conducted a retrospective, comparative cohort study of 200 patients who underwent bariatric surgery [Laparoscopic Sleeve Gastrectomy (LSG) or Roux-en-Y Gastric Bypass (RYGB)] at our department between January 2018 and January 2024.

#### 2.1.1 Inclusion criteria

(1) Age 18–40 years; (2) Patients whose primary caregivers were their parents or who resided with parents; (3) Complete follow-up data for at least 24 months.

#### 2.1.2 Exclusion criteria

(1) History of severe psychiatric disorders (e.g., schizophrenia); (2) Secondary causes of obesity; (3) Loss to follow-up.

### 2.2 Group allocation

Patients were assigned to two groups based on the timeframe of their surgery, reflecting a clinical protocol change.

#### 2.2.1 Control group (*n* = 100)

Patients treated between January 2018 and March 2021. They received standard surgical care and routine nutritional follow-up.

#### 2.2.2 Observation group (*n* = 100)

Patients treated between April 2021 and January 2024. They received standard surgical care plus a structured parental psychological intervention program. The rationale for introducing this intervention in 2021 was cumulative clinical observation suggesting an adverse impact of parental psychopathology on long-term weight outcomes.

### 2.3 Parental psychological intervention

The intervention was led by a multidisciplinary team and consisted of a structured 8-session program, each lasting 60 min, delivered in an individual family format.

#### 2.3.1 Assessment session (Session 1)

Parents were assessed using the Symptom Checklist-90 (SCL-90) and a validated Feeding Style Questionnaire (FSQ).

#### 2.3.2 Cognitive restructuring (Sessions 2–4)

Therapists worked with parents to identify and challenge maladaptive beliefs (e.g., “Feeding my child is the only way to show love”).

#### 2.3.3 Behavioral and boundary training (Sessions 5–7)

Strategies were employed to help parents establish healthy boundaries, reduce controlling behaviors, and foster the patient's autonomy regarding dietary choices. Homework tasks were assigned.

#### 2.3.4 Integration and relapse prevention (Session 8)

A consolidation session focused on maintaining changes and managing future challenges.

#### 2.3.5 Intervention fidelity

Interventions were delivered by two licensed clinical psychologists who followed a standardized manual. Case supervision bi-weekly and audio-recording of sessions with random reviews ensured fidelity to the protocol. No parents formally refused or dropped out of the therapy.

### 2.4 Outcome measures

Patients were followed up at 6, 12, and 24 months postoperatively.

#### 2.4.1 Primary outcome

Percentage of excess weight loss (%EWL).

#### 2.4.2 Secondary outcomes

Weight regain rate (defined as >15% increase from nadir weight), and dietary adherence scores.

#### 2.4.3 Psychometric measures

Reduction in parental control scores (measured by the FSQ) from baseline to the end of the intervention period. The dietary adherence score was a composite measure based on a validated self-report questionnaire administered at follow-up visits (Food Frequency and Adherence Questionnaire, FFAQ).

### 2.5 Statistical analysis

Continuous variables are presented as mean ± standard deviation (SD) and compared using independent samples *t*-tests. Categorical variables were compared using Chi-square or Fisher's exact tests. Pearson correlation analysis was used to assess the relationship between changes in parental control scores and dietary adherence. A *P*-value < 0.05 was considered statistically significant. Given the quasi-experimental design, we acknowledge that these analyses do not control for unmeasured confounders.

## 3 Results

### 3.1 Baseline characteristics

A total of 200 patients were included. Baseline demographics, preoperative BMI, and comorbidity rates were well-matched between groups, with no statistically significant differences (Table 1).

**Table 1:** Baseline characteristics of patients (*N* = 200).

Variable	Control group (*n* = 100)	Observation group (*n* = 100)	*P*-value
Age (years, Mean ± SD)	28.5 ± 6.2	29.1 ± 5.8	0.496
Gender (Male/Female, *n*)	45/55	42/58	0.667
Preoperative BMI (kg/m^2^, Mean ± SD)	42.3 ± 5.1	41.8 ± 4.9	0.492
Comorbidities, *n* (%)			
Type 2 Diabetes	32 (32.0%)	35 (35.0%)	0.663
Hypertension	45 (45.0%)	48 (48.0%)	0.671
OSAHS	28 (28.0%)	30 (30.0%)	0.752
Surgical Procedure, *n* (%)			
LSG	85 (85.0%)	88 (88.0%)	0.545
RYGB	15 (15.0%)	12 (12.0%)	0.545
Parental SCL-90 Anxiety Score (Mean ± SD)	2.8 ± 0.5	2.9 ± 0.6	0.223

### 3.2 Weight loss outcomes

**Figure 1:**
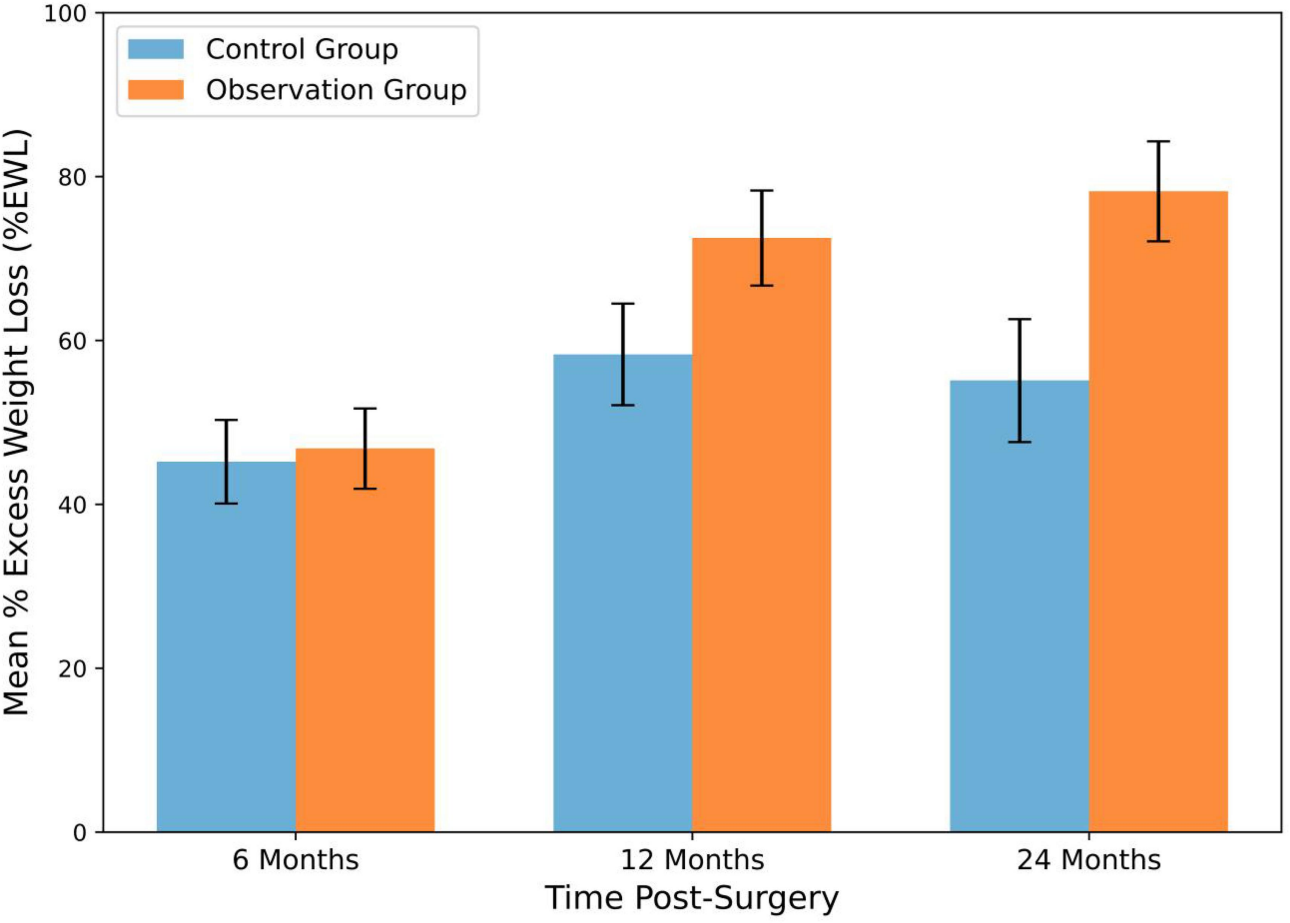
Comparison of percentage of excess weight loss (%EWL) between the observation and control groups at 6, 12, and 24 months post-bariatric surgery. Error bars represent standard deviation (**P < 0.001).

At 6 months postoperatively, %EWL was comparable between groups (Control: 45.2% ± 5.1% vs. Observation: 46.8% ± 4.9%, *P* = 0.32). By 12 months, a significant difference emerged (Observation: 72.5% ± 5.8% vs. Control: 58.3% ± 6.2%, *P* < 0.001). This difference persisted and widened at 24 months (Figure 1). The Observation Group demonstrated a mean %EWL of 78.2% ± 6.1%, significantly higher than the Control Group's 55.1% ± 7.5% (*P* < 0.001). Notably, the weight regain rate at 24 months was significantly lower in the Observation Group [4.2% (4/96)] compared to the Control Group [18.5% (17/92)] (*P* < 0.01).

### 3.3 Correlation analysis

**Figure 2:**
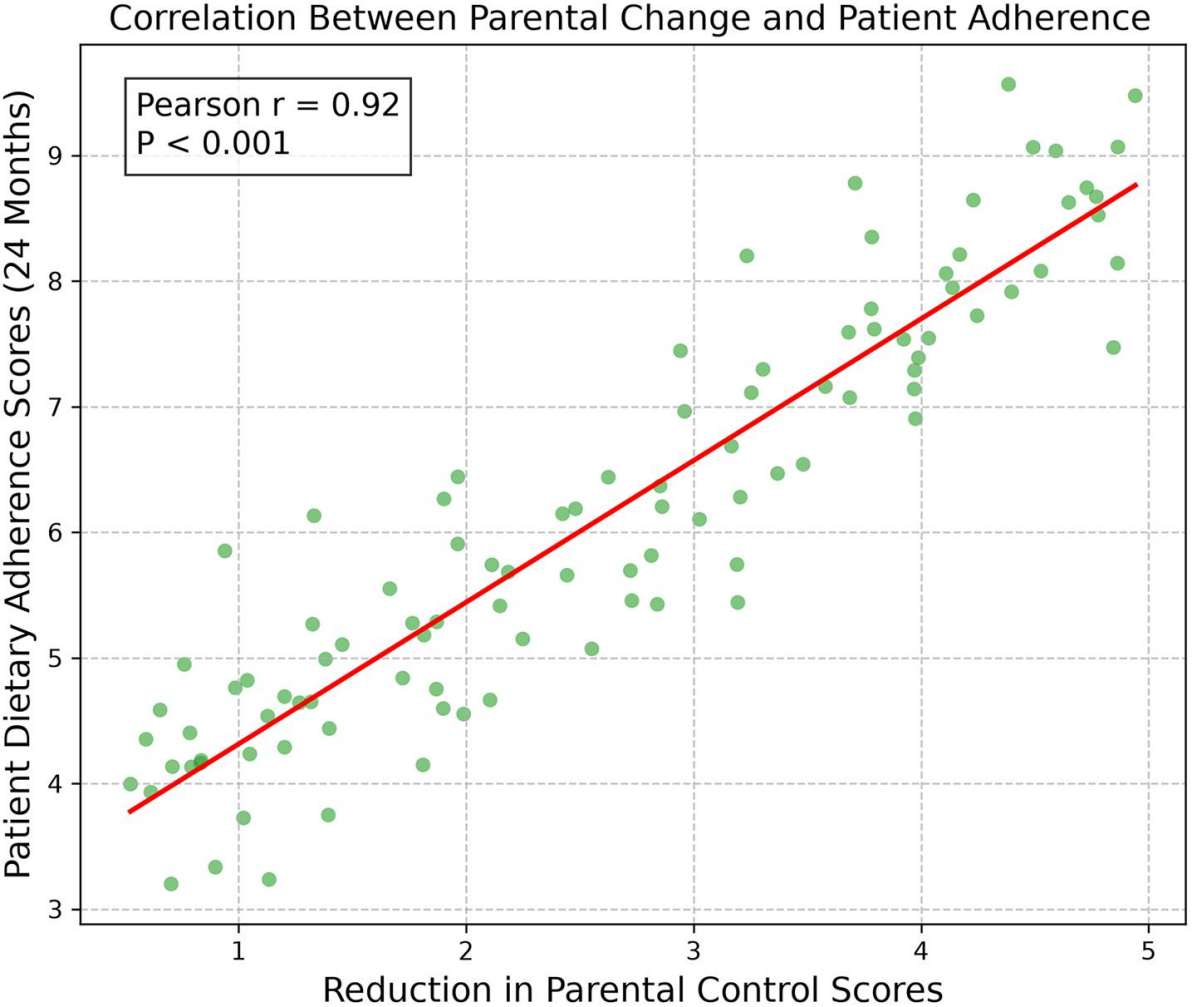
Pearson correlation between the reduction in parental control desire scores and patient dietary adherence scores at 24 months postoperatively in the observation group (*n* = 100).

In the Observation Group, a strong positive correlation was found between the reduction in parental control scores (from the FSQ) and patient dietary adherence scores at 24 months (r = 0.86, *P* < 0.001) (Figure 2). This indicates that a greater reduction in controlling parental behavior was associated with better adherence to the post-surgical diet.

## 4 Discussion

This retrospective study highlights a crucial, often overlooked factor in bariatric surgery: the psychological health of the patient's family system, particularly their parents. Our findings suggest that when bariatric surgery is combined with a structured psychological intervention for parents, patients achieve superior and more sustained weight loss. The results support the Family Systems Theory, positing that an individual's symptoms (obesity) can be a manifestation of dysfunctional family dynamics (8). In our cohort, parents exhibited “emotional eating” promotion driven by their own anxiety, a classic manifestation of anxiety projection (9). The phenomenon of “parental control” appears particularly detrimental post-surgery. Surgery imposes a mechanical limit on intake but does not eliminate psychological drive. If parents exert high control, patients cannot develop internal satiety cues and self-regulation (10). Our intervention, which reduced parental control and anxiety, effectively facilitated a “psychological weaning,” allowing patients to reclaim dietary autonomy.

Cultural factors are pertinent. In Chinese culture, food is often a primary love language, and parents maintain high involvement in adult children's lives (11). This makes targeting parents especially relevant in our setting. The significant improvement in the Observation Group (78.2% %EWL at 24 months) contrasts with the Control Group (55.1%), which is on the lower end of published benchmarks for these procedures. This suggests that unaddressed family psychopathology may be a cause of suboptimal outcomes in standard care, reinforcing the value of the intervention. The strong correlation between reduced parental control and improved patient adherence (r = 0.86) provides further support for the hypothesized mechanism.

However, we acknowledge several significant limitations. The *quasi-experimental design* (groups from different time periods) introduces the potential for temporal confounding, such as changes in surgical technique, follow-up protocols, and the impact of the COVID-19 pandemic. The intervention's non-randomized nature means causal inference cannot be established. This is a core limitation that tempers our conclusions. The psychological assessments relied on self-reported scales, subject to bias. Finally, the sample size, while sufficient for initial analysis, limits generalizability. Future prospective, randomized controlled trials (RCTs) are warranted.

## 5 Conclusion

The presence of parental psychopathology, especially anxiety and excessive control, appears to be a significant barrier to durable success after bariatric surgery. Incorporating structured psychological interventions for parents into the standard bariatric protocol is associated with markedly improved long-term weight loss outcomes. A holistic approach that treats the “family unit” is essential for optimizing outcomes of metabolic surgery.

## Data Availability

The original contributions presented in the study are included in the article/Supplementary Material, further inquiries can be directed to the corresponding author.
